# Extraction Procedure, Characteristics, and Feasibility of *Caulerpa microphysa* (Chlorophyta) Polysaccharide Extract as a Cosmetic Ingredient

**DOI:** 10.3390/md19090524

**Published:** 2021-09-18

**Authors:** Meng-Chou Lee, Han-Yang Yeh, Wen-Ling Shih

**Affiliations:** 1 Department of Aquaculture, National Taiwan Ocean University, Keelung City 20224, Taiwan; mengchoulee@email.ntou.edu.tw (M.-C.L.); 20833001@mail.ntou.edu.tw (H.-Y.Y.); 2 Center of Excellence for Ocean Engineering, National Taiwan Ocean University, Keelung City 20224, Taiwan; 3 Center of Excellence for the Oceans, National Taiwan Ocean University, Keelung City 20224, Taiwan; 4 Department of Biological Science and Technology, National Pingtung University of Science and Technology, 1, Shuefu Rd., Pingtung 91201, Taiwan

**Keywords:** *Caulerpa*, polysaccharides, anti-inflammation, moisture capacity, wound healing

## Abstract

The green alga *Caulerpa microphysa*, which is native to Taiwan, has a relatively high economic value and a well-developed culture technique, and is used mainly as a foodstuff. Its extract has been shown to exhibit antitumor properties, but the polysaccharide content of the extract and its anti-inflammatory and wound-healing effects and moisture-absorption and -retention capacity remain unknown. Hence, the objective of this study was to evaluate the potential of the polysaccharides in *C. microphysa* extract (CME) for use in cosmetics. The overall polysaccharide yield from the CME was 73.93% *w*/*w*, with four molecular weight fractions. The polysaccharides comprised 59.36 mol% mannose, 27.16 mol% glucose, and 13.48 mol% galactose. In addition, the CME exhibited strong antiallergic, wound-healing, transdermal-delivery, and moisture-absorption and -retention effects. In conclusion, the results suggested that CME potentially has anti-inflammatory and wound-healing effects and a good moisture capacity, which can be used in cosmetic applications.

## 1. Introduction

The commercial production of marine algae has been rapidly increasing over recent decades, whether by harvesting from natural resources or by cultivation. The application of algae is regarded as environmentally friendly, healthy, and sustainable for human beings [1]. Algae contain many compounds that can be used as foodstuffs, cosmetics, medicines, and pharmaceuticals, and they can also be used in aquaculture and agriculture [1]. This is because algae contain biochemical compounds such as pigments, lipids, cellulose, minerals, and polysaccharides, which have anticarcinogenic, anti-pigmentation, anti-dermatitis, emollient, humectant, antioxidant, anti-inflammatory, whitening, and anti-aging properties [2]. Among these compounds, a family of polysaccharides has been regarded as the most potentially effective for anti-inflammatory and wound-healing treatments [3]. Previous studies have revealed that their biological activity may be affected by their molecular weight, monosaccharide composition, polysaccharide dose concentration, and antioxidant content [4,5,6].

Notably, the extraction process is crucial for obtaining polysaccharides [7]. In general, hot water is considered the optimal solvent for the extraction of polysaccharides, and is often combined with autoclaving, microwaving, and ultrasonication [4,7]. These methods and instrumentation may not only affect the final extracted yield but also play a role in the overall economic cost [8,9]. Additionally, the crushing or milling, drying, and conservation process may also influence the extraction efficiency. In spite of the importance of these aspects, however, little information has been reported regarding the optimal extraction strategy for algae [4,7].

Furthermore, the structure of polysaccharides is complicated, which usually consists of various monosaccharides and esters. Thus, clarifying the structures and compositions of polysaccharides is crucial to comprehend the characteristics and potential functions of the objective sample [10]. In current, a series of approaches to methods for analyzing structures and compositions of polysaccharides have been reported. Acidic hydrolysis which involves acids such as HCl and H_2_SO_4_ is generally used to release the monosaccharides [10,11]. Liquid chromatography (LC), nuclear magnetic resonance (NMR), gas chromatography (GC), and mass spectrometry (MS) are usually used to analyze monosaccharides [10,12,13]. Notably, 2,3-naphthalene-diamine (NADA) can be used for the derivatization of aldoses and α-ketoacid-type to increase the efficiency for analyses [12]. Fluorescent monosaccharide labeling for NMR can increase the accuracy in identifying the monosaccharides and structure [10]. Those methods above are useful to optimize the qualitative and quantitative analysis of polysaccharides.

Inflammatory responses, also known as allergies, are induced by the degranulation of mast cells, which is an abnormal symptom associated with the overreaction of the immune system [13]. This response is debilitating, causing asthma, rhinitis, dermatitis, and other clinical allergy symptoms [14]. Wound healing involves the regeneration and replacement of connective tissue, and this process is frequently accompanied by inflammatory or other potentially injurious reactions. More specifically, wound healing is defined as the migration and proliferation of dermal and epidermal cells to fill or cover the wound, which is a dynamic process of tissue remodeling [15]. Notably, some people exhibit adverse reactions or need much longer recovery periods as a result of inflammatory inhibition of wound healing because of disease or a weakened immune system. Fortunately, previous studies have demonstrated the curative effects of some anti-inflammatory and wound-healing–accelerating substances extracted from plants, especially the polysaccharides found in marine algae [2,16]. Because of the similar chemical and biological properties of algal polysaccharides and mammalian glycosaminoglycan, the former are considered to contribute to immunoregulation in mammals [17]. However, although algae share similar polysaccharide structures, compound functions are determined by additional features such as chemical composition, molecular weight, and position on the polymer backbone. Thus, each algal species should be evaluated individually, because of their high degree of complexity and differing bioactive compounds [6].

The green alga *Caulerpa microphysa* (Weber Bosse) Feldmann 1955, also known as sea grape, is native to the intertidal zones in Taiwan, Japan, China, and the Philippines. It has a high economic value and a well-developed culture technique [18]. This alga consists mainly of carbohydrates (up to 70% *w*/*w*, data not shown), and is mainly used as a foodstuff, as feed, as aquarium algae, and for water-quality control, and it is popular as a traditional food. Building on its well-established culture technique and stable biomass supply, previous research has confirmed its antitumor properties using polypeptides extracted from it [19]. Extracts of other algal species have also been found to have positive pharmacological effects, such as the anticoagulant and antioxidant effects of polysaccharides extracted from *C. cupressoides*, the antioxidant effects of polysaccharides extracted from *C. prolifera* [20], the antiviral effects of the crude extract of *C. taxifolia* [21], and the antiproliferative effects of the crude extract of *C. racemosa* [22]. Although multiple functional properties have been demonstrated, there have been no reports on the anti-inflammatory and wound-healing properties of algae, particularly with regard to *C. microphysa*.

To further develop the pharmacological applications of *C. microphysa*, the objective of this study was to first develop the optimal polysaccharide extraction conditions for cultured *C. microphysa*, and then to identify and analyze the polysaccharides. Next, we hypothesized that the polysaccharide-rich extract of *C. microphysa* (CME) contains potentially useful bioactive compounds, and investigated the anti-inflammatory, wound-healing, and moisture-retention effects of various doses of CME. We expected to find that these polysaccharides could provide natural, healthy, safe, and effective raw materials for cosmetics.

## 2. Results and Discussion

### 2.1. Effects of Drying, Milling, and Extraction Procedures, and the Freeze-Drying Preservation of the Polysaccharide Yield

It is crucial to optimize extraction procedures based on the objectives of production [7]. In general, polysaccharide yield is positively correlated with both temperature and reaction period, but increasing these factors increases costs in terms of time and energy. However, the current situation may be changing as a result of the microwave–ultrasound extraction method, which has been shown to increase extraction efficiency for the microalga *Scenedesmus obliquus*, using lipid extraction, and for the red alga *Porphyra haitanensis*, using water-based extraction, but its efficiency with regard to green macroalgae remains unknown [4,7,23]. In addition, identifying the best drying and cell-disruption methods is crucial for optimizing the extraction process and decreasing the cost in terms of energy and time to achieve large-scale productivity [23,24].

**Figure 1:**
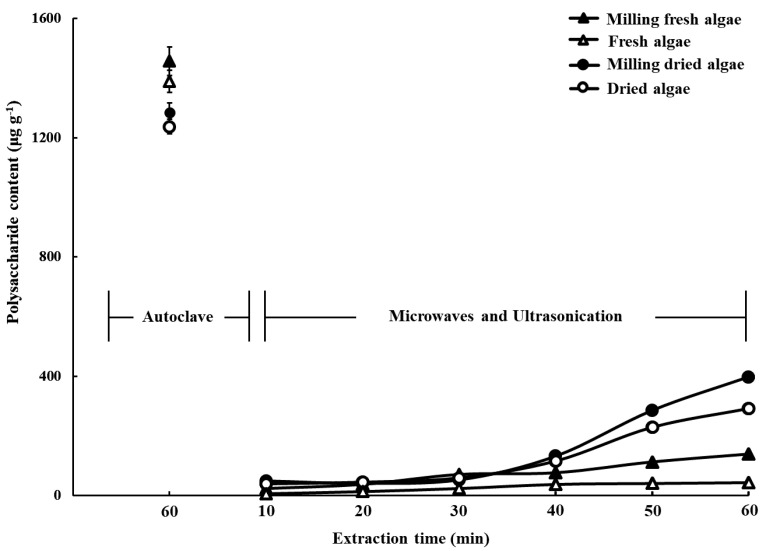
Effect of different pretreatments (none, milling, oven-drying, and both milling and oven-drying) and extraction procedures (autoclave and microwave–ultrasound) on the polysaccharide yield of water-based extraction of *Caulerpa microphysa*. Bars indicate SD, *n* = 3 (One-Way ANOVA and Scheffe’s a posteriori test).

The effects of the drying, milling, and extraction procedure used in this study on the polysaccharide yield are presented in Figure 1. Three factors significantly affected polysaccharide yield. The autoclave method was significantly more efficient than the microwave–ultrasound method. Both drying and milling improved extraction efficiency when using the microwave–ultrasound method, but only drying had any effect when using the autoclave method. Our result was different from those obtained for *Scenedesmus obliquus* and *Porphyra haitanensis* in previous studies, since we found that the extraction efficiency of the microwave–ultrasound method was lower than that of the autoclave method. This may be due to the use of different pretreatments and solvents [4,7]. Ansari et al., (2015) used lipid as a solvent to extract microalgae *S. obliquus* after cellulose was hydrolyzed by using H_2_SO_4_, and found that compared with autoclave, ultrasonication can obtain more reduced sugar [7]. The result may be due to the hydrolyzed cell wall, and the better thermal conductivity of lipid than water. Thus, lipid solvent can penetrate the sample and heat production to extract reduced sugar under ultrasonication adequately [8]. Whereas *C. microphysa* possessed an intact cell wall, which meant that the water solvent was hard to contact the algae sufficiently. Simultaneously, the heat production could not sufficiently destroy the inner cell wall tissue and release the polysaccharides under microwave–ultrasound extraction method. By comparison, the autoclave method was more effective in destroying cell walls than microwave–ultrasound extraction method by providing high thermal and pressure directly and then releasing polysaccharides from cells in this study.

**Figure 2:**
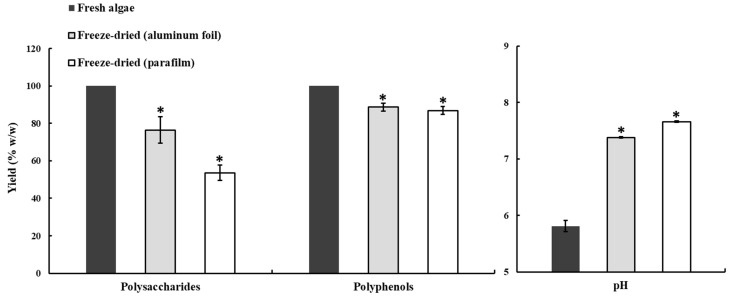
Effect of different freeze-drying covers (aluminum foil and parafilm) on the polysaccharide and polyphenol yield and the substrate pH. Bars indicate SD, *n* = 3, * indicates a significant difference (*p* < 0.05) compared to control (Student’s *t*-test).

Drying and milling improved the polysaccharide yield when followed by the microwave–ultrasound procedure, which suggests that the cell disruption resulting from this step aided the reaction of the solvent and heat transfer. However, drying resulted in a lower polysaccharide yield than just milling fresh samples when the autoclave method was used. It is thus likely that a loss of polysaccharides occurred during the drying process. In consideration of these findings, we prepared the CME from milled fresh algae, using the autoclave method, for use in further experiments with respect to freeze-drying preservation of the extract, the polysaccharide and polyphenol yield from samples kept with both types of cover was lower than that from the control samples. The polysaccharide recovery rate when using the aluminum foil and parafilm was 76.38 ± 7.06% and 53.64 ± 4.07% of the yield from the control samples, respectively, and the polyphenol recovery rate was 88.70 ± 2.07% and 86.88 ± 2.13%, respectively. In addition, after the freeze-drying and restoration processes, the pH of the samples covered with aluminum foil was 7.38 ± 0.61 and of those covered with parafilm was 7.66 ± 0.15, while that of the control was 5.81 ± 0.1 (Figure 2). Due to the parafilm cover having better resiliency than aluminum foil, thus we could make smaller holes by pin to avoid dried powder spraying during vacuum relief. These results illustrate that using the best freeze-drying procedure and material to cover the extract is important for preserving the polysaccharides.

### 2.2. Monosaccharide, Bioactive Ingredient, and Molecular Composition of CME

**Figure 3:**
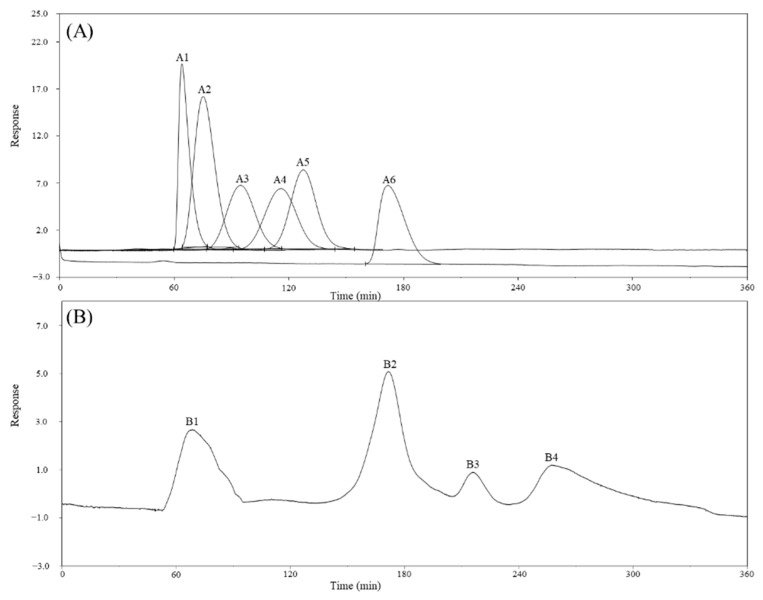
Molecular-weight distribution of (**A**) molecular-weight markers and (**B**) *Caulerpa microphysa* polysaccharide-rich extract (CME). Elution profile of standard pullulans showed A1–A5 are the curves for molecular-weight markers with weights of 100, 50, 20, 10, 5 kDa, respectively, and A6 indicates the curve for a glucose marker with a weight of 180 Da. B1–B4 are the four spectra obtained for CME using gel filtration chromatography with a Sephacryl S-200 High Resolution HiPrep 16/60 column as an eluent at a flow rate of 0.6 mL min^−1^ using a degas pump.

Previous studies have shown that biochemical composition, molecular weight, and chemical structure can affect biological activity [25]. Therefore, a deeper investigation of biochemical compounds is imperative. In this study, the in-depth analysis of the CME showed that it provided a total polysaccharide yield of 1457.17 ± 48.25 μg g^−1^, comprising 73.4% *w*/*w* of the biomass weight. These saccharides mainly comprised mannose (59.36 mol%), glucose (27.16 mol%), and galactose (13.48 mol%) (Table 1). Additionally, the extract provided a polyphenol yield of 16.62 ± 5.39 μg g^−1^ (Table 2). The molecular weight of the CME was determined via gel filtration chromatography using a refractive index detector system, which revealed four fractions (B1–B4) in the CME. Based on calibration with molecular-weight markers, the apparent molecular weight of B1 was 50–100 kDa, while that of the B2 fraction was similar to that of glucose, with an approximate molecular weight of 180 Da. The molecular weights of the B3 and B4 fractions were less than that of glucose and beyond the range of the molecular-weight markers (Figure 3).

**Figure 4:**
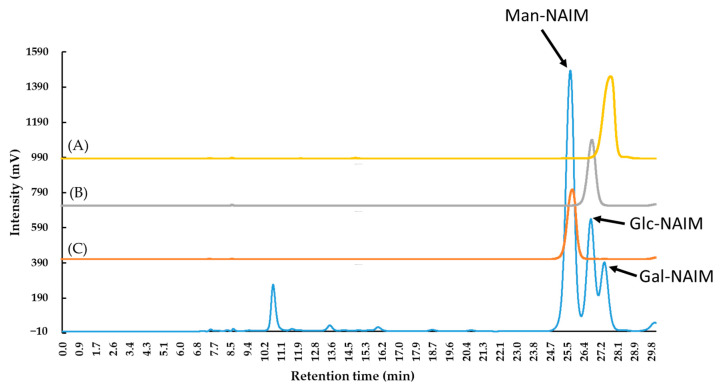
The HPLC-UV spectra of (**A**) Galactose naphthimidazole (Gal-NAIM) derivative, (**B**) Glucose naphthimidzaole (Glc-NAIM) derivative, (**C**) Mannose naphthimidazole (Man-NAIM) derivative, and (**D**) *Caulerpa microphysa* polysaccharide-rich extract (CME) obtained by gel filtration chromatography with a Biosil ODS-W 250 × 4.6 mm 5 μ (C18) column as an eluent at a flow rate of 0.4–1 mL min^−1^ using a gradient pump (Chromaster 5160, HITACHI, Tokyo, Japan).

**Figure 5:**
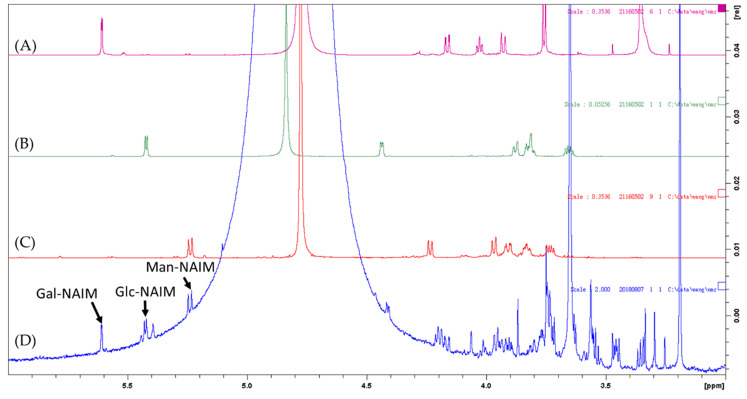
^1^H NMR spectra of (**A**) Galactose naphthimidazole (Gal-NAIM) derivative, (**B**) Glucose naphthimidzaole (Glc-NAIM) derivative, (**C**) Mannose naphthimidazole (Man-NAIM) derivative, and (**D**) *Caulerpa microphysa* polysaccharide-rich extract (CME).

Comparing our findings with previous research, the total polysaccharide yield with respect to biomass in this study was higher than that of most other water and organic extracts, such as *Nannochloropsis oculata* at 19% *w*/*w* [6]; *Ulva lactuca* at 26.01–28.29% *w*/*w* [5]; *Parachlorella* spp. at 73.8% *w*/*w* [26]; *Chondrus verrucosus* at 46.2–65.9% *w*/*w* [27]; and *Nostoc commune* at 82.2–84.6% *w*/*w* [28]. The results of this study suggest the feasibility of using *C. microphysa* as a cash crop to produce polysaccharides. Furthermore, the monosaccharide composition of the extract obtained in this study was different from that reported previously for species in the *Caulerpa* genus. For example, *C. brachypus* consists mainly of rhamnose, xylose, and glucose [11]; *C. racemose* consists of glucose (56.8 mol%) and galactose (31.8 mol%) [29]; and *C. cupressoides* consists mainly of galactose, mannose, and xylose [2]. Although all of these are in the same genus, they differ in their monosaccharide compositions. We also observed that the analysis systems used on the above *Caulerpa* genus are completely different, but all of them showed the monosaccharide compositions efficiently, e.g., the monosaccharides of *C. brachypus* hydrolyzed using H_2_SO_4_ were analyzed by GLC [11]; the monosaccharides of *C. racemose* hydrolyzed using HCl were analyzed by GC [29]; the monosaccharides of *C. cupressoides* hydrolyzed using HCl were analyzed by HPLC-RI [20] (Table 3). Notably, the monosaccharides in our study were obtained using HCl hydrolysis, and analyzed by both ^1^H NMR and HPLC-UV after Sugar-NAIM derivatization, and these two instruments showed the same monosaccharides composition (Figure 4 and Figure 5). The mentions above illustrated the diversity of the techniques which are beneficial to develop the studies in the carbohydrates field.

Previous studies have been summarized reported that the glycosidic bonds of *Caulerpa* genus including 4-Linked xylose, 6-linked galactose, 4-linked mannose, 6-linked α-D-mannopyranose, and 4-linked and 2-linked α-D-mannopyranose [30]. However, due to the information on the structure of the polysaccharides are not enough, the critical mechanism and function are not clarified [31]. Our result found the most monosaccharide in *C. microphysa* was mannose and followed were glucose and galatose. Although the monosaccharide composition and has been determined, its detailed glycosidic bonds were not clear. Hence, our further study will focus on investigating the potential glycosidic bond’s effect on the bioactivity of *C. microphysa*.

### 2.3. In Vitro β-Hexosaminidase Secretion Inhibition Assay

Allergies are a common health problem, affecting approximately 20% of the global population [32,33]. Antiallergic compounds usually act on mast cells, which are known to play a major role in the immediate type of allergic reaction. The signaling pathway involves binding of the IgE receptor to antigens, followed by the induction of degranulation in mast cells, which results in the release of chemical mediators including histamine, leukotrienes, and prostaglandins [34]. To evaluate the degranulation process resulting from an allergic reaction, RHB-2H3 cells, which are mucosal mast cells, can be used [35]. Moreover, β-hexosaminidase functions as a critical inflammatory mediator and is released along with histamine upon mast cell degranulation [36]. Thus, analysis of β-hexosaminidase secretion is widely used to evaluate the level of mast cell degranulation.

**Figure 6:**
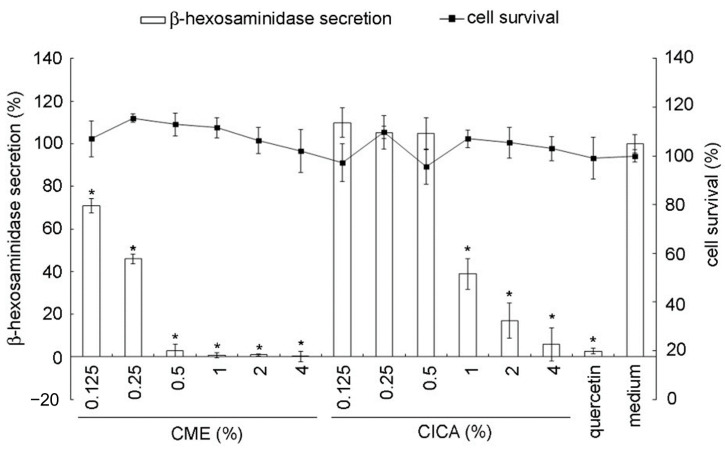
Antiallergic activity of *Caulerpa microphysa* polysaccharide-rich extract (CME) in RBL-2H3 cells. Bars indicate SD, *n* = 3 * indicates a significant difference (*p* < 0.05) compared to the medium control (Student’s *t*-test). CICA: *Centella asiatica*.

As shown in Figure 6, antigen-mediated signaling causes a critical increase in the secretion of β-hexosaminidase. However, CME showed a superior concentration-dependent inhibitory. When 0.25% of CME were added, the β-hexosaminidase inhibition rates were more than 50%. When dose of CME more than 0.5%, β-hexosaminidase release was almost suppressed completely. Previous in vitro studies reported that the hot water extract of *Ecklonia cava* and *Chrymenia wrightii* have more than 50% degranulation inhibit at a dose of 100 µg mL^−1^, in addition, MeOH extract of *Petalonia binghamiae*, *Scytosiphone lomentaria*, *Undaria pinnatifida*, *Porphyra dentata*, *Codium fragile*, and *Ulva japonica* have more than 50% degranulation inhibit at a dose of 200 µg mL^−1^, illustrate that extract solvent significantly effect on degranulation inhibit [14]. Maruyamam et al., (2005) use in vivo study to show that mekabu fucoidan at a dose of 50 µg mL^−1^ significantly decreased the type 2 T-helper cytokines, IL-4, IL-5, and IL-13 level, which are the chemical mediators to induce degranulation [37]. Overall, our results suggest that effective degranulation inhibitory activity occurs. Compared to CICA extract, which possesses well-known antiallergic activity and is widely used in commercially available products, the antiallergic potential of CME was better at the same concentration.

### 2.4. In Vitro Wound-Healing Activity Assay

Healthy, intact, wound-free skin can prevent dehydration, microorganisms and irritants as an important protective way of individual. The in vivo wound healing process is tightly controlled by multiple growth factors released at the wound site, such as Platelet-derived growth factor (PDGF), Transforming Growth Factor (TGF), and Epidermal growth factor (EGF). Some natural products have been used extensively in wound care with excellent effects [38] To determine the wound re-epithelialization potential of CME, an artificial and uniform cellular scar was created. TGF-β, a growth factor, stimulates matrix protein production and induces faster healing, and was therefore included as a positive control [39].

**Figure 7:**
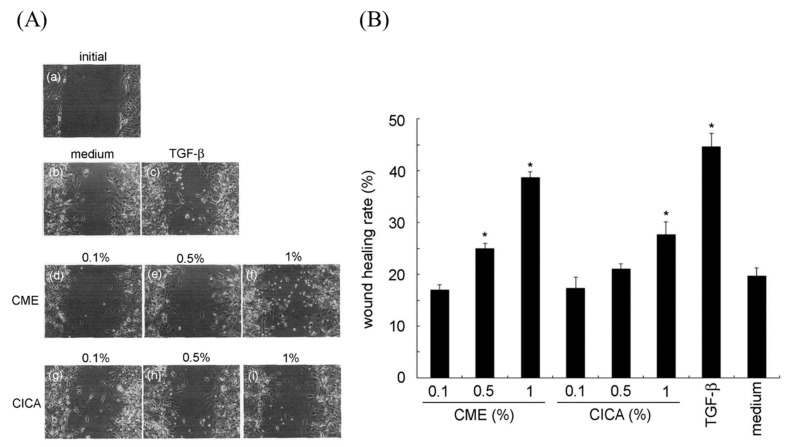
Effects of *Caulerpa microphysa* polysaccharide-rich extract (CME) on wound healing. (**A**) Microscope imaging to evaluate wound healing using confluent 3T3-L1 fibroblasts. Representative images showing the initial scratch (wound) at t = 0 (a), 8 h after CME treatment (d–f) and CICA treatment (g–i) with indicated concentration. Cell migration into the wound area was observed. A single representative area is shown 8 h after various treatments. 10 ng/mL TGF-β was used as the positive control. (**B**) The wound-healing percentage 8 h post-treatment is shown on the *y* axis, normalized against the initial time (0 h), for each treatment condition. Bars indicate SD, *n* = 3, * indicates a significant difference (*p* < 0.05) compared to the medium control (Student’s *t*-test). CICA: *Centella asiatica*.

The cellular wound healing after 8 h of treatment is illustrated in Figure 7A. Compared to the untreated medium control, treatment of 3T3-L1 cells with 0.5% CME for 8 h significantly improved the wound-healing response: wound repair following the 0.5% and 1% CME treatments increased by approximately 25 and 39%, respectively (Figure 7B). CICA exhibited significant wound-healing activity at a 1% concentration. Previous study report that aqueous extract of Spirulina platensis can significantly induce cell migration of HDF cells, which is better than methanolic, ethanolic and allantoin extract [15]. The similar result also observes to methanolic extract of *Moringa oleifera* inhibit wound healing [40].

Above reports explain that the solvent may the key factor effect on the proliferation of migrated cell and to confirm the feasibility by using algae extract as resource for wound healing. The potential function of wound healing may be due to the glycosaminoglycan (GAG) extract from algae mediates cellular interaction and growth factor signaling pathways associated with wound recovery process [41,42]. Although not compare multiple solvent extracts on the effect of wound-healing here, however, we clearly demonstrated the feasibility of using hot-water extract of *C. microphysa* to induce the wound-healing activity of 3T3-L1 fibroblasts. Overall, these results suggest that CME had comparable healing effects.

### 2.5. Hydroxyproline Production and In Vitro Permeation Assay

The process of skin aging is mainly due to the slowing down of the proliferation of keratinocytes in the epidermis and fibroblasts in the dermis, resulting in the reduction of extracellular matrix of the dermis, including collagen, elastin, proteoglycans, glycosaminoglycans. Among these molecules, collagen is considered the most important [43]. Currently, mechanisms underlying the loss of collagen in aging or damaged skin have not been fully delineated. It is known that collagen synthesis is highly regulated by genes. Previous studies have found the plant-derived compounds with anti-oxidant properties can restore fibroblast function through modulation of signaling pathways like MAPKs and NF-κB [3].

**Figure 8:**
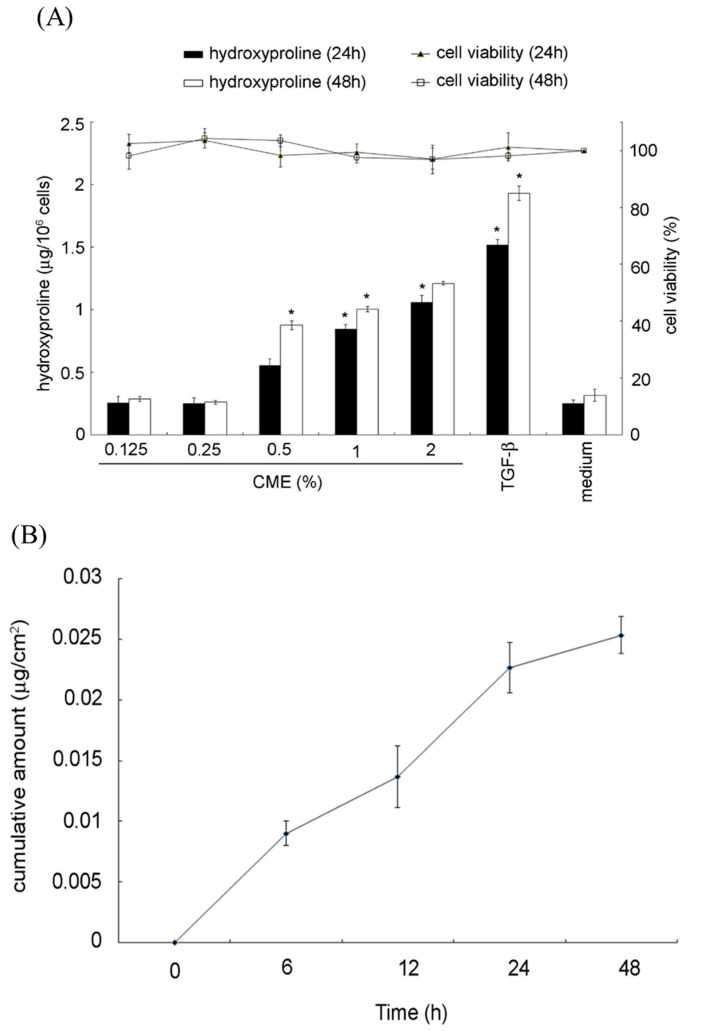
(**A**) *Caulerpa microphysa* polysaccharide-rich extract (CME) enhanced hydroxyproline production in a dose-dependent manner. Bars indicate SD, *n* = 3 (Student’s *t*-test), * indicates a significant difference (*p* < 0.05) compared to the medium control. 10 mg mL^−1^ TGF-β was included as the positive control and for comparison. (**B**) Cumulative amount of CME that permeated through the Strat-M membrane over time.

Hydroxyproline is the compound produced upon digestion of collagen and elastin, and it has been suggested that it may be able to improve aged or damaged skin. In order to evaluate the potential anti-wrinkle properties of CME, an in vitro assay to determine hydroxyproline production was performed. Hydroxyproline concentrations increased in CME-treated hydrolyzed cellular lysates (Figure 8A) relative to the medium control. Cell viability was not affected by treatment condition.

To further evaluate the potential of CME for use as a cosmetic raw material, its transdermal-delivery efficacy was measured using a synthetic membrane engineered to mimic human skin. This assay aids in predicting diffusion efficiency into human skin for a wide range of materials and compounds. The cumulative total membrane permeation by the CME at various time points is shown in Figure 8B. As can be seen, CME clearly shows potential in terms of cumulative skin permeation.

Few studies have been conducted to evaluate the penetration of raw cosmetics materials, mostly focused on the potential functional proteins on wound response [44]. However, the collagen-producing fibroblasts are located in the skin dermis, thus, effective cosmetics ingredients should be positively correlative with skin penetration. Strat-M^®^ synthetic membrane has similar function on either human cadaver skin or Strat-M^®^ membrane, which has been regarded as an ideal material for skin penetration investigating.

The factors such as permeability coefficient (Kp), lag time, skin deposition, and molecular size are reported to be relative to the epidermis penetration [45]. Skin tissue plays a crucial role in filtering the entry of substances. As illustrated in a clinical research, the permeability of the epidermis restricted the potential of chemical compounds as drugs, since only compounds with molecules of less than 500 Da can penetrate the skin [46]. According to Figure 3B, fraction B2 of the main polysaccharides of CME, with molecules of 180 Da, can pass through the epidermis theoretically. In addition, compared to the lipophilic substances, the hydrophilic substance is more efficient when passing through the stratum corneum barrier. Therefore, we assumed that the water-soluble CME may mainly penetrate the stratum corneum through channels including sweat glands and hair follicles. But, since the two pathways mentioned (sweat glands and hair follicles) only occupy a small ratio of the skin surface area [47], we believe that CME may also penetrate the skin through other channels. In conclusion, CME possessed excellent skin penetration properties, and the critical mechanism worths further exploration.

### 2.6. Moisture Absorption and Retention Assay

It’s well known that bio-polysaccharides in cosmetics function as gelling agents, viscosity adjuster, thicker as well as water-holding by means of its swelling capacity [42]. These are due to the polysaccharides can solubilize in water and can also react with skin fibrin to form an extracellular gel matrix, result in moisturizing. Moisturizing is the most important function of skincare products. Effective moisturizing facilitates active moisture absorption and retention by the skin [48]. The moisture absorption and moisture retention properties of CME were therefore assessed and compared with those of several cosmetics on the market in this study.

**Figure 9:**
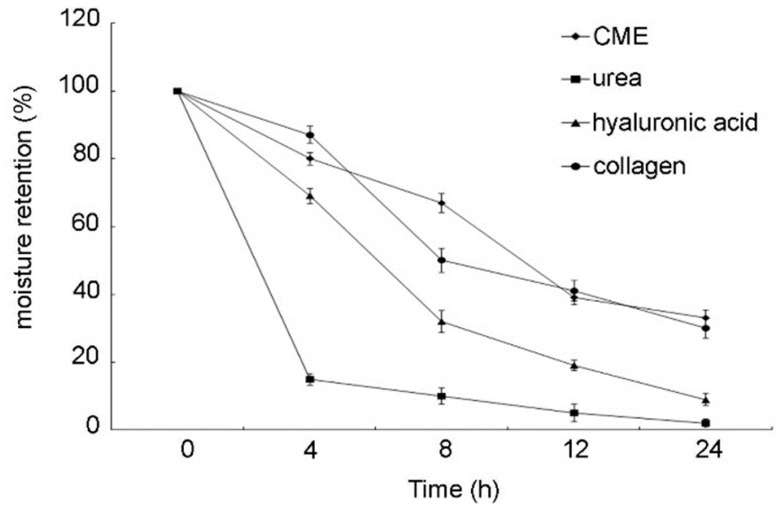
Moisture retention of *Caulerpa microphysa* polysaccharide-rich extract (CME) samples at 35 °C and 75% relative humidity. Bars indicate SD, *n* = 3 (One-Way ANOVA and Scheffe’s a posteriori test).

The moisture absorption results at various time points and humidity levels are shown in Table 4. In terms of water-absorption capacity, CME was better than collagen, similar to hyaluronic acid, and poorer than urea. The moisture-retention capacity over 24 h is shown in Figure 9, and that of CME was excellent; far better than that of collagen and hyaluronic acid, and similar to that of urea.

The aspect of moisture absorption capacity, when at 81–84% relative humidity, the water extract of *C. microphysa* showed the optimized water-absorption capacity (78%) than water extract of *Nostoc sphaeroides* (29.9–32.5%) [49], water extract of *Enteromorpha prolifera* (47.58–61.39%) [50], and *Sargassum horneri* (7.4–9.5%) [51]. On the other hand, the aspect of moisture- retention capacity, the water extract of *C. microphysa* showed the water-absorption capacity at 40%, which was lower than water extract of *N. sphaeroides* (55.6–58.2) [49], *E. prolifera* (89.50–91.80%) [50], and *S. horneri* (49.1–53%) [51].

Due to the moisture-absorption/retention abilities are effect by complicated biochemistry compounds especially in molecular weight and sulfated content [52]. Thus, further analyzed the molecular weight among the above species, the molecular weight of *N. sphaeroides* extract was 199–99 kDa [49], *S. horneri* extract was 179 kDa to 21.42 kDa [51], *E. prolifera* extract was 147 kDa to 44.8 kDa [50], and *C. microphysa* extract was 100 kDa to <50 kDa. Although the different molecular weight levels in each species, but seem no correlation with the moisture-absorption/retention abilities. Despite, our result demonstrated that CME had high potential and product applicability and could be expected to become a novel multifunctional moisturizer.

## 3. Materials and Methods

### 3.1. Cultivation Conditions

The *C. microphysa* used in this study was isolated from the intertidal zone in northeastern Taiwan. The algae were washed with 1.5% povidone–iodine and 2 μm-filtered UV-irradiated sterilized seawater to remove any adhering debris or epiphytic organisms. They were then cultivated in a 1 t fiberglass tank under a 12:12 h light:dark regime. The tank was aerated and maintained at an irradiance level of 80–100 μmol photons m^−2^ s^−1^, which was measured using a Lighting Passport spectrometer (ALP-01, Asensetek, New Taipei City, Taiwan). The seawater was refreshed every three days, and 20 g t^−1^ ammonium sulfate was added to ensure healthy growth conditions. When the mass of the algae reached 2 kg, they were harvested for the experiments.

### 3.2. Pretreatment and Extraction

#### 3.2.1. Extraction of Polysaccharides

To detect the effect of pretreatment on the polysaccharide content, the biomass of algae was divided into four treatments as follows: (1) fresh algae; (2) milled fresh algae; (3) oven-dried algae; and (4) milled oven-dried algae. Two extraction methods were then investigated: (a) autoclave extraction (SS320, Tominaga, Taipei City, Taiwan), and (b) microwave–ultrasound extraction (EXTRACTOR 200, IDCO, Marseille, France). The fresh and dry treatments were performed using a 1:1 and a 1:0.06 (*v*/*v*) mixture, respectively, of the algae with distilled water in a 1 L serum bottle, the fresh and dry treatments were performed using a 1:1 and a 1:0.06 (*v*/*v*, taking moisture loss into consideration) mixture, respectively, of the algae with distilled water in a 1 L serum bottle. at 40 °C for 2 d. The fresh and dried algae were milled with a blender (Blendtec, Orem, UT, USA) or a pulverizer at room temperature, respectively. Before extraction, treatments (1) and (2) were stored at −20 °C in a freezer, while (3) and (4) were placed in a Moisture-Proof Box (EDRY, Taichung, Taiwan).

At the extraction step, the autoclaved samples were treated at 121 °C, 1.5 lbs for 60 min. The microwave–ultrasound samples were treated with sonication at 100 mv and microwaves at 1000 W and mixed in the microwave–ultrasound extractor for 60 min, and 20 mL samples were extracted every 10 min. After extraction, all samples were centrifuged at 15,000× *g* for 10 min (CR21G, Hitachi, Tokyo, Japan), after which the supernatant was filtered through a sterile 0.22 μm filter membrane (Sartorius, Chöttingen, Germany). The filtered supernatant was freeze-dried into powder (FD10/-80, FIRSTEK, New Taipei, Taiwan), then analyzed for total polysaccharide yield. The optimal extraction conditions were then used for further analysis and in vitro studies.

#### 3.2.2. Analysis of Total Polysaccharide Content

To determine the total polysaccharide content, 1 mg of lyophilized powder was dissolved in 20 mL of ultrapure water and analyzed using the phenol-sulfuric acid method [53].

#### 3.2.3. Analysis of Sugar Composition

We assessed the polysaccharide content and sugar composition of the extracted polysaccharide powder using a polysaccharide component assay kit from SugarLight (New Taipei City, Taiwan) following the method of Lin et al. (2010) [54]. A 1 mg sample of purified and dried polysaccharides was added to 1.0 mL of hydrolysis solution and the resulting mixture was stirred for 2 h at 80 °C, then dried using a vacuum pump. The resulting powder was mixed well with 2 mg 2,3-naphthalenediamine, 1 mg iodine, and 1 mL acetic acid, then stirred at room temperature for 1 h to achieve fluorescent monosaccharide labeling. After drying the solvent, we quantified and qualified the monosaccharide-naphthylimidazole via ^1^H NMR spectrometry (Bruker AV600, Rheinstetten, Germany) and via HPLC-UV (Hitachi L2130 pump with UV L2420).

#### 3.2.4. Analysis of Molecular Weight

The molecular weight of the polysaccharides was determined via high-performance liquid chromatography on a high-resolution gel filtration column (HiPrep 16/60 Sephacryl-S-200 HR column, Merck, Darmstadt, Germany) with ultrapure water at a flow rate of 0.6 mL min^−1^, detected using a refractive index detector, and visualized using Chromatography Workstation software (EChrom Data System v1.0, Lixing Technology, Hsinchu city, Taiwan).

#### 3.2.5. Analysis of Polyphenols

To determine the polyphenol content, 1 mg of lyophilized powder was dissolved in 20 mL of ultrapure water and analyzed using the Folin–Ciocalteu method [55].

#### 3.2.6. Analysis of Preservation Losses

To analyze ingredient loss during the freeze-drying process, we evaluated the effects of two different covers on the total polysaccharide and polyphenol content of the extracts. Briefly, 20 mL of the extraction mixture was added to sterile 50 mL centrifuge tubes and stored at −80 °C for 48 h. Next, aluminum foil or parafilm was used to cover the tube mouth, and the samples were freeze-dried immediately. After 72 h, the samples were taken out and injected with an equal volume of distilled water by weight. The total polysaccharide and polyphenol content were then analyzed and compared to that of the control samples.

### 3.3. Analysis of In Vitro Immunostimulatory Activity

#### 3.3.1. Reagents

We prepared *C. microphysa* polysaccharide-rich extract (CME) in our laboratory. Quercetin, TGF-β, and 3-(4,5-dimethylthiazol-2-yl)-2,5-diphenyl-2H-tetrazolium bromide (MTT) were obtained from Sigma (St. Louis, MO, USA). *Centella asiatica* (CICA) was purchased from Giga Fine Chemical (Taipei, Taiwan). Urea and collagen were purchased from Shunyi Chemical (Taichung, Taiwan), and hyaluronic acid was obtained from Chengyi Chemical (Taipei, Taiwan).

#### 3.3.2. Analysis of MTT Cytotoxicity

Cytotoxicity was evaluated via MTT assay according to ISO10993-5. RBL-2H3 cell lines were grown in the required medium and seeded onto a 96-well plate at 5 × 10^3^ cells per well until adherence. Thereafter, the medium was removed, treated with the indicated samples at the indicated concentrations, and further incubated for a range of durations. The MTT was then added and cleaved with mitochondrial reductase to form formazan crystals. The purple formazan was solubilized by adding dimethyl sulfoxide (DMSO), and the optical density (OD) was read at 570 nm, with a reference wavelength of 690 nm, using a microreader (Thermo Scientific, Waltham, MA, USA).

#### 3.3.3. Analysis of Sensitization and Stimulation for Degranulation

RBL-2H3 cells were grown in Eagle’s minimal essential medium (MEM) containing 4 mM L-glutamine, 1.5 g L^−1^ sodium bicarbonate, 0.1 mM nonessential amino acids, 1 mM sodium pyruvate, and 15% heat-inactivated fetal bovine serum (FBS). Cells were seeded at 10^5^ cells per well in a 24-well plate. After adherence for 24 h, the cells were sensitized by adding 0.5 μg mL^−1^ anti-DNP IgE (Sigma) for 24 h, washed twice with Siraganian buffer, and incubated with Siraganian buffer for 10 min. They were then treated with CME or CICA and incubated for 2 h. Subsequently, 10 μg mL^−1^ of antigen DNP-BSA was added, and the samples were incubated for 20 min to stimulate degranulation. Quercetin was used as a positive control by followed the method of Mlcek et al., (2016) [56]. The β-hexosaminidase activity was quantified via a colorimetric reaction using substrate 4-nitrophenyl N-acetyl-β-D-glucosaminide (Sigma) according to the method used by Quah et al., (2020) [57].

#### 3.3.4. Analysis of Wound Healing

This experiment was performed by the Industrial Technology Research Institute (Hsinchu, Taiwan). Mouse embryo fibroblast 3T3-L1 (BCRC60159) cells were cultured in Dulbecco’s modified Eagle medium (DMEM) containing 4 mM L-glutamine, 1.5 g L^−1^ sodium bicarbonate, 4.5 g L^−1^ glucose, and 10% calf serum. Cells were seeded onto a SPLScar Block (SPL Life Sciences, Seongnam, Korea) at a density of 2 × 10^5^ cells well^−1^ on a 24-well plate. After adherence for 24 h, the blocks were removed and incubated with medium containing either a test sample or the positive control TGF-β for an additional 8 h. The wound area was photographed and the percentage wound-healing rate was calculated as (A_0_ − A_8_)/A_0_, where A_0_ was the wound area at 0 h and A_8_ was the wound area at 8 h. Image analysis was performed using ImageJ software v1.8.0_112.

#### 3.3.5. Analysis of Hydroxyproline

Human skin fibroblast CCD966SK cells (BCRC60153) were grown in MEM in Earle’s balanced salt solution (BSS) containing 0.1 mM nonessential amino acids, 1.5 g L^−1^ sodium bicarbonate, 1 mM sodium pyruvate, and 10% FBS. The cells were seeded on a 24-well plate at 2 × 10^5^ cells well^−1^ overnight to ensure adherence, then incubated with either CME at various concentrations or the positive control, TGF-β. Hydroxyproline was measured using a commercially available kit (Biovision, Milpitas, CA, USA) according to the manufacturer’s instructions. Absorbance was determined at 560 nm using an ELISA reader (Thermo Scientific).

#### 3.3.6. In Vitro Permeation Studies

In vitro percutaneous absorption was measured using a manual diffusion system (PermeGear, Riegelsville, PA, USA) equipped with Strat-M membrane (Merck), which is a well-established synthetic model for transdermal diffusion testing. The 25 mm Strat-M membrane was mounted between the donor and receptor compartments and secured tightly with clamps. The available area of the membrane was 0.635 cm^2^. A 20 mg mL^−1^ solution of CME was loaded in the donor compartment, and the receptor compartment was filled with phosphate-buffered saline (PBS). The diffusion cells were placed on a magnetic stirring block, and the receptor compartment was maintained at 37 °C using a circulating water bath. Aliquots of 200 μL were withdrawn from the receptor compartment at various time points up to 48 h and analyzed using a total carbohydrate assay kit (Biovision) to determine the amount of CME that had permeated through the Strat-M membrane.

#### 3.3.7. Analysis of Moisture Absorption and Retention Capacity

Both moisture absorption and moisture retention capacity were analyzed according to the method used by Song et al., (2019) [58].

### 3.4. Statistical Analysis

Data were analyzed using Microsoft Excel 2010 and IBM SPSS Statistics 22.0 (IBM Corp, Armonk, NY, USA). One-way analyses of variance (ANOVAs) were used to test for the significance of differences between pretreatments, water extraction procedures, and moisture retention. Student’s *t*-test was used to analyze freeze-drying efficiency, β-hexosaminidase inhibition, wound healing, hydroxyproline production, and cell viability. Where significant differences were identified by the ANOVAs, we used Scheff’e Test to compare the means across the treatment conditions. All data are presented as the means ± standard deviation (SD) of three independent experiments, with each experiment performed at least in triplicate. A *p*-value < 0.05 was considered statistically significant.

## 4. Conclusions

In this study, we described an effective extraction and preservation strategy for CME and performed an analysis of its polysaccharide compositions and molecular weights. We then demonstrated that CME has sufficient safety, antiallergic, and wound-repair properties; enhances hydroxyproline production; and is able to penetrate a Strat-M membrane and accumulate over time. Furthermore, CME possesses excellent moisture-absorption and -retention properties and can aid in the prevention of skin aging in multiple ways. Overall, *Caulerpa microphysa* has high potential for use in the cosmetics industry.

## Figures and Tables

**Table 1:** Polysaccharide composition of *Caulerpa microphysa* polysaccharide-rich extract (CME) analyzed using a nuclear magnetic resonance spectrometer.

Total Saccharides % (*w*/*w*)	Monosaccharide Composition mol%		
	Mannose	Glucose	Galactose
73.4	59.36	27.16	13.48

*w*/*w*: Monosaccharide weight as a percentage of total saccharide weight.

**Table 2:** The polysaccharide and polyphenol yield of *Caulerpa microphysa* polysaccharide-rich extract (CME).

Polysaccharides (mg g^−1^)	Polyphenols (mg g^−1^)
1457.17 ± 48.25	16.62 ± 5.39

**Table 3:** Comparison of analysis systems and monosaccharides compositions in the *Caulerpa* genus.

Species	Analysis Systems	Monosaccharides Composition	References
*Caulerpa microphysa*	HCl hydrolysis, Sugar-NAIM* derivatization, ^1^H NMR* and HPLC*-UV analysis	Total sugar (*w*/*w*): 73.4% Mannose 59.36%, Glucose 27.16%, Galactose 13.48%	This study
*C. brachypus*	H_2_SO_4_ hydrolysis, GLC* analysis	Rhamnose, Xylose, Glucose	Lee et al., 2004 [11]
*C. racemosa*	HCl hydrolysis, GC* analysis	Total sugar (*w*/*w*): 36–53.7% Uronic acid (*w*/*w*):3.9–7.9% Neutral sugar (Glucose 56.8%, Galactose 31.8%, Mannose 11.4%)	Ji et al., 2008 [29]
*C. cupressoides*	HCl hydrolysis, HPLC-RI* analysis	Total sugar (*w*/*w*): 52.38–59.60% Galactose, Glucose, Mannose, Xylose, Rhamnose, Fucose	Costa et al., 2012 [20]

*NAIM: Naphthimidazole; *NMR: Nuclear Magnetic Resonance spectroscopy; HPLC: High-performance liquid chromatography; *GLC: Gas-liquid chromatography; *GC: Gas Chromatography; *RI: Refractive Index.

**Table 4:** Moisture-absorption capacity of various sorbents.

Relative Humidity (%)	Sample	% Moisture Absorption at Given Time Point (h)			
		8	12	24	48
32	CME	0	0	2	6
	urea	0	2	4	10
	hyaluronic acid	0	0	2	2
	collagen	0	0	0	0
75	CME	2	17	42	72
	urea	0	22	68	87
	hyaluronic acid	0	20	39	59
	collagen	0	6	6	7
84	CME	9	18	60	78
	urea	20	56	99	119
	hyaluronic acid	2	29	78	95
	collagen	2	3	9	19

## Data Availability

Not applicable.
